# RELATIONSHIP BETWEEN SCHOOLCHILDREN’S LEVELS OF PHYSICAL ACTIVITY, ANTHROPOMETRIC INDICES AND PULMONARY FUNCTION

**DOI:** 10.1590/1984-0462/2021/39/2019189

**Published:** 2020-06-22

**Authors:** Fernanda Pazini, Caroline Pietta-Dias, Cristian Roncada

**Affiliations:** a Pontifícia Universidade Católica do Rio Grande do Sul, Porto Alegre, RS, Brazil.; b Universidade Federal do Rio Grande do Sul, Porto Alegre, RS, Brazil.

**Keywords:** Asthma, Spirometry, Motor activity, Anthropometry, Asma, Espirometria, Atividade física, Antropometria

## Abstract

**Objective:**

To evaluate and to correlate levels of physical activity with the pulmonary function of children with and without a diagnosis of asthma.

**Methods:**

This study was conducted in two phases with schoolchildren aged between eight and 16 years old in Porto Alegre/RS. In the first phase (cross sectional), the sample was classified as asthmatic if a physician had ever diagnosed them with asthma and if they reported symptoms and treatment for the disease in the past 12 months. In the second phase (control-case), the following were measured: anthropometry, physical activity levels, time spent in front of screens, and lung function (spirometry). Data are presented in mean and standard deviation or median and interquartile interval and by absolute and relative values. Chi-square, Student’s t-test or Mann-Whitney test and Spearman correlation were applied, with p<0.05 being significant.

**Results:**

605 students participated in the study, 290 children with a clinical diagnosis of asthma and 315 classified as a control. 280 (47.3%) were male children, with an average age of 11.0±2.3 years old. The spirometric values showed differences in the classifications of airway obstruction levels between the asthma and control groups (p=0.005), as well as in the response to bronchodilator use for FEV1/FVC (p=0.023). In the correlation assessment, there was no correlation between physical activity with anthropometric values, nor with pulmonary function, pre-and post-bronchodilator.

**Conclusions:**

The study demonstrates that there is no relationship between either anthropometric values or physical activity levels with pulmonary function of asthmatic children.

## RESUMO

**Objetivo:**

Avaliar e correlacionar os níveis de atividade física com a função pulmonar de crianças com e sem diagnóstico de asma.

**Métodos:**

Estudo realizado em duas fases, em escolares de oito a 16 anos de Porto Alegre (RS). Na fase I (transversal), classificaram-se como asmáticos os escolares com diagnóstico positivo de um médico alguma vez na vida, com crises e tratamento para a doença nos últimos 12 meses. Na fase II (caso controle), foram avaliados: antropometria, níveis de atividade física e tempo gasto em frente às telas e função pulmonar (espirometria). Os dados são apresentados por média e desvio padrão ou mediana e intervalo interquartil e por valores absolutos e relativos, sendo aplicados os testes χ^2^, *t* de Student ou de Mann-Whitney e correlação de Spearman, com valor de significância p<0,05.

**Resultados:**

Participaram do estudo 605 escolares, 290 crianças com diagnóstico clínico de asma e 315 classificadas como controle. Do total, 280 (47,3%) crianças eram do sexo masculino, com média de idade de 11,0±2,3 anos. Os valores espirométricos demonstraram diferenças nas classificações dos níveis de obstrução das vias aéreas entre grupos asma e controle (p=0,005), além da resposta ao uso de broncodilatador, para o volume expiratório forçado no primeiro segundo (VEF_1_)/capacidade vital forçada (CVF) (p=0,023). Não houve correlação entre a prática de atividades físicas e valores antropométricos, tampouco entre a função pulmonar e o pré e pós-uso de broncodilatador.

**Conclusões:**

O estudo demonstrou não existir relação entre valores antropométricos e níveis de atividade física com a função pulmonar de crianças asmáticas em idade escolar.

## INTRODUCTION

Asthma is a chronic disease with a high prevalence, especially in children. As such, it is considered a worldwide public health problem. It has several severity classifications, and only the correct diagnosis can indicate the best therapeutic method to be applied.^1^ Generally, its diagnosis is made from clinical findings, with acute episodes, symptoms between crises, a personal and family history of the disease, evolution of the disease, reduced lung function and response to treatment.^1^ ^,^ ^2^

Physical exercise can exacerbate the symptoms. However, induced bronchoconstriction must be understood as separate from uncontrolled symptoms during a crisis, and as such there different treatments.^3^ Exercise-induced bronchoconstriction is transient and affects more than 40% of the child population.^4^ Hyperventilation during physical activity can dry out and cool the airways, triggering an acute crisis.^5^ However, asthmatic children doing regular physical activities show reduced levels of systemic inflammation, and thus it is widely recommended by specialists as part of prophylactic treatment.^6^ ^,^ ^7^

The recommendations of the American College of Sports Medicine (ACSM) suggest that children and young people practice at least 60 minutes of physical activity daily.^8^ Activities must be at a moderate to vigorous level, in order to have an increase in cardiorespiratory frequency.^8^ Studies show that the young population in general has very low levels of fitness and physical activity, causing an increase in the incidence of overweight and obesity cases in the child population.^9^ ^,^ ^10^ ^,^ ^11^ Being overweight and obese reduces lung capacity and volume.^12^ Among these changes, one of the most important is the decrease in functional residual capacity (FRC).^13^ When an individual is obese, their FRC is lower due to compression in the rib cage, reducing the dimensions of the region because of the high amount of fat mass. The diaphragm moves higher due to the distention in the abdomen.^14^ In order for normal lung function to perform in terms of its normal capacity and volume, the respiratory system must also be working in harmony.^14^

Asthmatic and obese children have their symptoms exacerbated, as they have a decreased response rate to inhaled corticosteroids.^10^ Some epidemiological studies assume that asthma and obesity are correlated.^11^ ^,^ ^12^ Based on these facts, the objectives of the present study were to assess and correlate the levels of physical activity with the pulmonary function of school children with and without a diagnosis of asthma.

## METHOD

A study was carried out in two phases with students from public schools in Porto Alegre (RS) aged 8 to 16 years old:

- Phase I (cross-sectional): a characterization of the sample, with an abbreviated questionnaire applied to the guardians of the students, following the standards of the International Study of Asthma and Allergies in Childhood (ISAAC) study.^15^ It contained four questions for the identification of children with asthma and healthy children: 1) Has the student ever experienced wheezing in the chest or shortness of breath?; 2) Has the student ever had a medical diagnosis of asthma or bronchitis?; 3) In the last 12 months, did the student show wheezing or shortness of breath?; 4) In the last 12 months, did the student ever use medication for asthma or bronchitis?
- Phase II (control case): after characterization and identification of the asthma and control (healthy) groups, the outcomes of the anthropometry, levels of physical activity and lung function (spirometry) were evaluated.

Prior to inclusion in the study, both guardians and patients consented to participate with a free and informed consent form (guardians) and an agreement form (children/adolescents).

In addition, the study was approved by the Research Ethics Committees (CEP) of the Pontifícia Universidade Católica do Rio Grande do Sul, as well as by the Municipal Health Secretariat of Porto Alegre, under Substantiated Report No. 73,585/12 and No. 7,793/2012, respectively.

In the anthropometric evaluation, the following were evaluated:

- The body mass index (BMI),^16^ calculated by BMI=weight (kg)/stature^2^ (m) and demonstrated by the Z score.
- For the fat percentage (%F), following the methodology proposed by Slaughter et al.,^17^ skin folds (triceps and medial calf) were evaluated, using the formula (%F=0.735 (Σ2DC) +1.0) for boys and (%F = 0.610 (Σ2DC) +5.0) for girls, where Σ2DC equals the sum of the two folds.
- Waist to stature ratio (WSR),^18^ was calculated by dividing waist circumference with height (WSR=waist/stature ratio).

All of the anthropometric assessments were collected by a single, previously trained researcher. For the skin folds, a scientific adipometer of the brand/model Lange (Cambridge Scientific Industries, Inc., Cambridge, Maryland, United States) was used. Body mass was obtained with individuals in an orthostatic position, with minimal clothing, no shoes, and weighed using a digital scale (G-Tech, Glass 1 FW, Rio de Janeiro, Rio de Janeiro, Brazil) previously calibrated with a precision of 100 grams. Stature was measured with the participants barefoot, feet in a parallel position, ankles together, arms extended along the body and the head positioned so that the lower part of the eye socket was on the same plane as the external ear hole. Stature measurements were taken using a portable stadiometer (Altura Exata, TBW, São Paulo, São Paulo, Brazil) with an accuracy of 1 mm.

To assess lung function, spirometry tests were applied before and after the application of 400 µg of salbutamol (bronchodilator), following international criteria and guidelines (American Thoracic Society and European Respiratory Society).^19^ The values of forced expiratory volume in the first second (FEV1), forced vital capacity (FVC) and the division between the two variables (FEV1/FVC) were collected. The best maneuver of at least three acceptable and two reproducible ones were recorded, according to international criteria. Like the BMI values, the spirometric parameters were adjusted for the target audience, and were presented by Z score.

For the purpose of assessing physical activity levels, a questionnaire designed and validated for the study population was applied. It was proposed by Hallal et al., ^20^ and was composed of extra-class physical activity questions and time spent in front of screens as a risk factor for physical inactivity. All questions corresponded to the week prior to the application of the questionnaire (last seven days). In order to characterize physically active or sedentary children, the total sum of activities equal to or greater than 10 minutes was used as a criterion, with the cutoff point being 300 minutes (<300 minutes=physically inactive/sedentary and ≥300 minutes=physically active). As for the physical inactivity risk, ≥2 hours/day for high risk of inactivity and <2 hours/day for low risk of physical inactivity were adopted.

For the design and sample calculation, both the selection of schools and that of students occurred in a randomized way (simple random). In total, seven public schools in Porto Alegre were selected, based on the sample calculation the ISAAC studies,^15^ which predicted a confidence level of at least 95% and sampling error of up to 5%. For phase I, 2,500 students were needed. For phase II, the sample size was estimated at 576 students (288 children with asthma and 288 ­control/­healthy children; 1:1).

For the statistical analysis, descriptive and categorical variables were presented by absolute and relative frequencies, and descriptions of continuous variables, were presented by means and standard deviation (M±SD) or median and interquartile (MD-IQ). To make comparisons between the groups (asthma and control), normality was assessed by the Z test (Kolmogorov-Smirnov), and the values were analyzed using the χ2 test and the t independent Student test for homogeneous variables and the Mann-Whitney test for non-homogeneous variables. In addition, for the purpose of correlating levels of physical activity, anthropometry and lung function, Spearman’s correlation was applied, with a significance value of p<0.05.

## RESULTS

In phase I, 2,899 questionnaires were distributed in seven public schools in the city in order to identify and characterize the sample. Of these, 2,500 were considered to be eligible for the study (86.2%), reporting an asthma prevalence rate of 20.4% (n=511). Of the 2,500 schoolchildren, 1,211 (48.4%) were male, and had an average age of 11.4±2.3 years.

In phase II, 605 students were randomly selected and evaluated, 280 (47.3%) of whom were male, and had a mean age of 11.0±2.3 years. In addition, students were divided into two groups:

- Asthma group: 290 students classified as asthmatic.
- Control group: 315 students classified as healthy.

In Table 1, the classifications of the sample are presented, by group.

**Table 1:** Characteristics of the students analyzed. Porto Alegre, RS, Brazi.

	Asthma group (n=290)		Control group (n=315)		p
	n (%)	X±SD	n (%)	X±SD	
Age*		11.0±2.3		11.0±2.3	0.915
Sex (female)**	150 (51.7)		172 (54.6)		0.798
Economic classification**					
Class A	2 (0.7)		1 (0.3)		0.112
Class B	51 (17.6)		83 (26.3)		
Class C	201 (69.3)		212 (67.3)		
Class D	32 (11)		17 (5.4)		
Class E	4 (1.4)		2 (0.6)		
Color/race**					
Caucasian	180 (62.1)		168 (53.3)		0.098
Black	47 (16.2)		63 (20)		
Light-skinned black	63 (21.7)		84 (26.7)		

n (%): absolute and relative values; X±SD: mean and standard deviation; *independent Student’s *t* test (scalar variables); **χ^2^ test.

In Table 2, there are pulmonary function values, anthropometric measurements and levels of physical activity for categorical variables. Spirometric values demonstrate differences in the classifications of airway obstruction levels (p≤0.005), as well as in the pre/post-bronchodilator assessment, with the group of asthmatics showing a higher frequency for response (> 12%) after using a bronchodilator (p≤0.023). As for anthropometric values, none of the three applied variables showed differences (BMI p≤0.424, %F p≤0.962, WSR p≤0.471). The same was considered for the levels of physical activity or risk of physical inactivity due to leisure time in front of screens.

**Table 2:** Lung function, anthropometry and levels of physical activity in the asthma (n = 290) and control (n = 315) groups.

	Asthma group n (%)	Control group n (%)	p-value
Lung function (spirometry)			
Normal	249 (87.1)	290 (93.8)	0.005
Light obstruction	36 (12.6)	19 (6.1)	
Moderate obstruction	1 (0.3)	-	
Baseline differences/post-BD (>12%)			
FEV_1_	48 (16.9)	39 (12.7)	0.150
FVC	23 (8.1)	18 (5.9)	0.286
FEV_1_/FVC	14 (4.9)	5 (1.6)	0.023
Anthropomorphic measurements			
*Body Mass Index (Z score)*			
Eutrophic	189 (65.1)	212 (67.3)	0.424
Overweight	67 (23.1)	61 (19.4)	
Obese	34 (11.7)	42 (13.3)	
*Fat Percentage (%F)*			
Eutrophic	127 (44.6)	139 (45.3)	0.962
Overweight	60 (21.0)	61 (19.9)	
Obese	98 (34.4)	107 (34.8)	
*Waist/stature ratio (WSR)*			
Low risk	260 (91.2)	285 (92.8)	0.471
High risk	25 (8.8)	22 (7.2)	
Levels of physical activity/inactivity			
Performed physical activity in the last 7 days	192 (66.2)	204 (64.8)	0.576
Commuted from home/school (on foot)	235 (81.0)	261 (82.9)	0.173
Levels of physical activity (active)	95 (32.8)	95 (30.2)	0.492
Time in front of screens (≥2 h/day)	239 (82.4)	257 (81.6)	0.792

BD: bronchodilator; FEV1: forced expiratory volume in the first second; FVC: forced vital capacity; FEV1 / FVC: Tiffeneau index (relationship between the two variables).

In Table 3, there are pulmonary function values, anthropometric measurements and levels of physical activity for categorical variables. The spirometric values reveal differences in the pre-use volumes of the bronchodilator (FEV1 and ­FEV1/­FVC, p = 0.006 and p <0.001, respectively). As for anthropometric values, only the WSR variable showed a difference between groups (p=0.021), with no discrepancies between physical activity measures and the risk of physical inactivity due to leisure time in front of screens.

**Table 3:** Lung function, anthropometry and levels of physical activity in the asthma (n = 290) and control (n = 315) groups.

	Asthma group		Control group		p -value
Lung function (pre-BD)	n	M±SD	n	M±SD	†
FEV_1_	286	0.8±1.5	309	1.1±1.4	0.006
FVC	286	0.9±1.5	309	1.0±1.4	0.475
FEV_1_/FVC	286	-0.2±1.1	309	0.1±1.1	<0.001
Lung function (post-BD)	n	M±SD	n	M±SD	†
FEV_1_	284	1.3±1.7	307	1.5±1.6	0.342
FVC	284	1.2±1.6	307	1.2±1.6	0.671
FEV_1_/FVC	284	0.2±1.1	307	0.5±1.1	0.001
Anthropomorphic measurements	n	MD(IQ)	n	MD(IQ)	‡
Body mass index¥	290	0.0(0.0-1.0)	315	0.0(0.0-1.0)	0.424
Fat percentage (%F)	285	19.0(16.9-22.1)	307	18.8(16.7-21.8)	0.985
Waist/stature ratio (WSR)	285	0.8(0.7-0.8)	307	0.4(0.4-0.5)	0.021
Physical activity (min/week)	290	160.0(50.0-382.5)	315	180.0(75.0-340.0)	0.973
Physical inactivity (h/week)	290	4.1(2.5-7.0)	315	4.2(2.4-6.7)	0.962
Television	283	19.0(10.0-29.0)	306	17(.0(9.0-28.0)	0.517
Videogames	113	12.0(5.5-22.5)	138	10.0(4.9-16.3)	0.058
Computer	164	8.0(4.0-20.0)	178	13.0(6.9-21.0)	0.125

M ± SD: mean and standard deviation; MD (IQ): median and interquartile; BD: bronchodilator; FEV1: forced expiratory volume in the first second; FVC: forced vital capacity; FEV1/FVC: Tiffeneau index (relationship between the two variables); † independent Student’s t test; ‡ Mann-Whitney test; ¥: BMI presented by Z score.

In Table 4A, there are the correlations between the levels of physical activity or risk for physical inactivity and anthropometric values, with no correlation existing between the variables studied. In Table 4B, the correlations between the practice of physical activity, the time spent in front of screens, and the increase or decrease in lung function are reported. These results suggest that there is no correlation between the time spent on physical activities and anthropometric measures, nor between lung function, in the pre- and post-use of bronchodilators, in either group.

**Table 4:** (A) Correlation between levels of physical activity and anthropometric indices; (B) correlation between levels of physical activity and lung function before and after use of bronchodilators.

(A)	Asthma group						Control group					
	SF (n=285)		BMI (n=290)		WSR (n=285)		SF (n=307)		IMC (n=315)		WSR (n=307)	
Physical activity levels¥	0.057		0.018		0.014		-0.065		-0.081		-0.008	
Risk for physical inactivity#	-0.089		0.029		0.002		0.048		-0.023		-0.047	
(B)	Asthma group						Control group					
	Pre-BD (n=286)			Post-BD (n=284)			Pre-BD (n=309)			Post-BD (n=307)		
	FEV_1_	FVC	FEV_1_/FVC	FEV_1_	FVC	FEV_1_/FVC	FEV_1_	FVC	FEV_1_/FVC	FEV_1_	FVC	FEV_1_/FVC
Physical activity levels¥	0.031	0.037	-0.026	0.059	0.040	-0.009	-0.109	-0.114	-0.040	-0.094	-0.090	-0.037
Risk for physical inactivity#	-0.045	-0.039	-0.019	-0.011	-0.018	0.004	0.029	0.017	-0.021	0.048	0.023	0.019

¥ In minutes per week; # hours a day in front of screens; SF: skin fold; BMI: body mass index; WSR: waist/stature ratio; BD: bronchodilator; FEV1: forced expiratory volume in the first second; FVC: forced vital capacity; FEV1/FVC: Tiffeneau index (relationship between the two variables).

## DISCUSSION

The objectives of this study were to evaluate and correlate the levels of physical activity with the pulmonary function of children with and without a clinical diagnosis of asthma. The results pointed to a non-existent correlation for both the group of asthmatics and the group of healthy children. In addition, both groups showed very similar values in terms of physical activity levels and anthropometric measurements, with differences only for pulmonary function (FEV1 and FEV1/FVC). These results indicate that students in this age group have very similar physical and anthropometric patterns, regardless of whether or not they have a chronic respiratory disease.

Regarding the spirometric indices presented, the results showed differential normality between the groups, since asthma, a chronic obstructive disease, leads to reduced values of FEV1 and FEV 1/FVC. ^21^ ^,^ ^22^ In addition, asthmatics tend to present differences between their spirometric values before and after the use of a bronchodilator (salbutamol), due to the obstructive pathophysiological condition, regardless of the severity of asthma.^23^ In other words, airway obstruction, even if mild, results in increased resistance to airflow, air retention in the airways and overuse of the inspiratory muscles, which leads to altered pulmonary function.^23^

The relationship between FEV1/ FVC corroborated the results found in FEV1, indicating obstructive disorders at rest (p≤0.001). Pulmonary function tests are important markers for the management of chronic respiratory diseases in adults and children, especially those with asthma. The analysis of changes in lung volumes and flows during forced expiration helps in the treatment and prognosis of the disease. Regular pulmonary function assessments are essential for monitoring children with asthma. There may be bronchial obstruction in an asthmatic child, even if he or she is asymptomatic. This represents a greater risk of exacerbating a severe crisis associated with reduced lung function.^24^

After the use of bronchodilators, there was an improvement in FEV1/ FVC in the group of students with asthma and in the control group (p≤0.003). The results were positive when there were differences in the spirometry results with the use of bronchodilators. Bronchodilators are the most widely used drugs for the treatment of lung diseases, especially in cases of asthma. They are usually applied by inhaling, as they act quickly.^25^ Continuing treatment with the use of inhaled corticosteroids reduces the frequency and severity of exacerbated asthma attacks, and the number of hospitalizations and emergency room visits. Appropriate treatment also improves quality of life, lung function and bronchial hyperresponsiveness, decreasing exercise-induced bronchoconstriction. Such substances must be used before beginning exercise, since relax the smooth muscle of the airways by acting on the autonomic nervous system receptors. Thus, when performing an activity, the individual’s crisis symptoms will be reduced or even nonexistent.^23^ ^,^ ^25^

With regard to anthropometric measurements, the continuous variables of the BMI data, fat mass index and waist/stature ratio are described. There was no significant difference in fat mass index for either group (p≤0.757). For the WSR, there was a discrepancy between the two groups (p≤0.048). The group of asthmatic children had WSR rates above those without asthma. However, previous studies do not confirm these findings,^15^ ^,^ ^26^ and may have been random discoveries, since BMI and skin folds did not show differences between the groups evaluated in the present study.

Physical exercise is considered one of the main palliative factors in the treatment of asthma. Asthmatic children may have their daily and physical activities reduced due to the exacerbation of symptoms.^27^ Exercise-induced bronchoconstriction that is associated with asthma or not, appears along or at the end of physical activity and is a transient limitation. Better disease control is linked to increased aerobic exercise.

One previous study that assessed functional capacity in children with asthma pointed out that 88% of asthmatics and 56% of healthy children performed less than two hours a week of physical activity. These numbers worsen when parents report in questionnaires that they believe physical activity is dangerous for their children, thinking that exercise triggers crises. ^28^ Another study also confirmed these data. Using questionnaires answered by parents, the authors observed that urban and school-aged asthmatic children are less active and that> 20% do not reach the goal of normal physical activity.

The parents’ reluctance to encourage exercise may be due to exercise-induced bronchospasm, which can often be as intense as an asthma attack. In families whose parents believe that exercise can improve asthma control and quality of life, children are more active.^29^ ^,^ ^30^

Data show that children aged 8-12 years spend an average of 6 hours a day involved with electronic devices. ^25^ Those who suffer from asthma may be more adept at using technology, due to their sedentary lifestyle, their condition of being overweight and not having the triggered symptoms. Our study showed no correlation between physical inactivity and the use of electronic media.

Our study presented several limitations. The population included urban school-age children and youth, most of whom were not asthmatic and only a small number who had moderate to severe asthma. In addition, the overweight/obesity group was small, limiting the sampling power of the associations. A longer study is suggested, in which assessments can be made in all seasons of the year.

Although the international guideline Global Initiative for Asthma (GINA),^1^ supported by the Brazilian Society of Pulmonology and Tisiology (*Sociedade Brasileira de Pneumologia e Tisiologia* - SBPT),^3^ provides specialized medical monitoring and adherence to pharmacological treatment for asthma control associated with the regular practice of physical activities to reduce body fat mass and improve the mechanisms of pulmonary function, our study did not show differences in the variables of levels of adherence to physical activity practices and anthropometric indices between children with and without a diagnosis of asthma.

## References

[1] Bateman ED, Hurd S, Barnes P, Bousquet J, Drazen J, FitzGerald M (2008). Global strategy for asthma management and prevention: GINA
executive summary. Eur Respir J.

[2] Pereira EA, Ferreira PR, Araújo ME, de Carvalho ST, Carvalho LN (2016). Comparative study of quality of life among patients with chronic
obstructive pulmonary disease and patients with asthma. Rev Ceuma Perspectivas.

[3] Sociedade Brasileira de Pneumologia e Tisiologia (2012). Diretrizes da Sociedade Brasileira de Pneumologia e Tisiologia
para o manejo da asma-2012. J Bras Pneumol.

[4] Lamar RA, Fonseca AA, Neves MA, Valença LM (2001). Cardiorespiratory response to incremental progressive maximal
exercise in asthmatic patients. J Pneumologia.

[5] Melo RE, Solé D (2007). Differential diagnosis of exercise induced asthma: a challenge
for the specialist. Rev Bras Alerg Imunopatol.

[6] Taketomi EA, Marra SM, Segundo GR (2005). Fisioterapia em asma: efeito na função pulmonar e em parâmetros
imunológicos. Fit Perf J.

[7] Wicher IB, Ribeiro MA, Marmo DB, Santos CI, Toro AA, Mendes RT (2010). Effects of swimming on spirometric parameters and bronchial
hyperresponsiveness in children and adolescents with moderate persistent
atopic asthma. J Pediatr (Rio J).

[8] Haskell WL, Lee IM, Pate RR, Powell KE, Blair SN, Franklin BA (2007). Physical activity and public health: updated recommendation for
adults from the American College of Sports Medicine and the American Heart
Association. Med Sci Sports Exerc.

[9] Ng M, Fleming T, Robinson M, Thomson B, Graetz N, Margono C (2014). Global, regional, and national prevalence of overweight and
obesity in children and adults during 1980-2013: a systematic analysis for
the Global Burden of Disease Study 2013. Lancet.

[10] Guedes DP, Guedes JE (2001). Esforços físicos nos programas de educação física
escolar. Rev Paul Educ Fis.

[11] Kêkê L, Samouda H, Jacobs J, Di Pompeo C, Lemdani M, Hubert H (2015). Body mass index and childhood obesity classification systems: A
comparison of the French, International Obesity Task Force (IOTF) and World
Health Organization (WHO) references. Rev Epidemiol Sante Publique.

[12] Weinmayr G, Forastiere F, Büchele G, Jaensch A, Strachan DP, Nagel G (2014). Overweight/obesity and respiratory and allergic disease in
children: international study of asthma and allergies in childhood (ISAAC)
phase two. PloS One.

[13] Winck AD, Heinzmann-Filho JP, Soares RB, Silva JS, Woszezenki CT, Zanatta LB (2016). Effects of obesity on lung volume and capacity in children and
adolescents: a systematic review. Rev Paul Pediatr.

[14] Assunção SN, Daltro CH, Sorte NC, Ribeiro HC, Bastos ML, Queiroz CF (2014). Lung function in the absence of respiratory symptoms in
overweight children and adolescents. J Bras Pneumol.

[15] Asher M, Weiland S (1998). The International Study of Asthma and Allergies in Childhood
(ISAAC). ISAAC Steering Committee. Clin Exp Allergy.

[16] Giugliano R, Melo AL (2004). Diagnosis of overweight and obesity in schoolchildren:
utilization of the body mass index international standard. J Pediatr (Rio J).

[17] Slaughter MH, Lohman T, Boileau R, Horswill C, Stillman R, Van Loan M (1988). Skinfold equations for estimation of body fatness in children and
youth. Hum Biol.

[18] Ribeiro-Silva RC, Florence T, Conceição-Machado ME, Fernandes GB, Couto RD (2014). Anthropometric indicators for prediction of metabolic syndrome in
children and adolescents: a population-based study. Rev Bras Saude Matern Infant.

[19] Arets H, Brackel H, van der Ent C (2001). Forced expiratory manoeuvres in children: do they meet ATS and
ERS criteria for spirometry?. Eur Respir J.

[20] Farias JC, Lopes AS, Mota J, Santos MP, Ribeiro JC, Hallal PC (2012). Validity and reproducibility of a physical activity questionnaire
for adolescents: adapting the Self-Administered Physical Activity
Checklist. Rev Bras Epidemiol.

[21] Sheehan WJ, Permaul P, Petty CR, Coull BA, Baxi SN, Gaffin JM (2017). Association between allergen exposure in inner-city schools and
asthma morbidity among students. JAMA Pediatr.

[22] Teach SJ, Gergen PJ, Szefler SJ, Mitchell HE, Calatroni A, Wildfire J (2015). Seasonal risk factors for asthma exacerbations among inner-city
children. J Allergy Clin Immunol.

[23] Reddel HK, Hurd SS, FitzGerald JM (2014). World Asthma Day. GINA 2014: a global asthma strategy for a
global problem. Int J Tuberc Lung Dis.

[24] Vidal P, Mattiello R, Jones M (2013). Spirometry in preschool children. Pulmão RJ.

[25] Tavares MG, Pizzichini MM, Steidle LJ, Nazário NO, Rocha CC, Perraro MC (2010). The asthma control scoring system: translation and cross-cultural
adaptation for use in Brazil. J Bras Pneumol.

[26] Lopes WA, Rosário N, Leite N (2010). Broncoespasmo induzido pelo exercício em adolescentes asmáticos
obesos e não obesos. Rev Paul Pediatr.

[27] Ostrowska-Nawarycz L, Wroński W, Błaszczyk J, Buczyłko K, Nawarycz T (2006). Bronchial asthma prevalence in children and youth with
overweight. Pol Merkur Lekarski.

[28] Brockmann P, Caussade S, Prado F, Reyes B, Viviani P, Bertrand P (2007). Physical activity and obesity in asthmatic
children. Rev Chil Pediatr.

[29] Lang DM, Butz AM, Duggan AK, Serwint JR (2004). Physical activity in urban school-aged children with
asthma. Pediatrics.

[30] Freitas PD, Silva RA, Carvalho CR (2015). Effects of exercise on clinical asthma control. Rev Med (São Paulo).

